# The sialic acid binding activity of the S protein facilitates infection by porcine transmissible gastroenteritis coronavirus

**DOI:** 10.1186/1743-422X-8-435

**Published:** 2011-09-12

**Authors:** Christel Schwegmann-Weßels, Sandra Bauer, Christine Winter, Luis Enjuanes, Hubert Laude, Georg Herrler

**Affiliations:** 1 Institute for Virology, University of Veterinary Medicine Hannover, Bünteweg 17, 30559 Hannover, Germany; 2 Clinic for Poultry, University of Veterinary Medicine Hannover, Bünteweg 17, 30559 Hannover, Germany; 3 Centro Nacional de Biotecnología, CSIC, Department of Molecular and Cell Biology, Campus Universitario de Cantoblanco, Darwin 3, 28049 Madrid, Spain; 4 Institut National de la Recherche Agronomique, Unité de Virologie Immunologie Moléculaires (VIM), Domaine de Vilvert, Jouy-en-Josas, 78350, France

**Keywords:** coronavirus S protein, sialic acid binding activity, TGEV, IBV, cultured cells

## Abstract

**Background:**

Transmissible gastroenteritis virus (TGEV) has a sialic acid binding activity that is believed to be important for enteropathogenicity, but that has so far appeared to be dispensable for infection of cultured cells. The aims of this study were to determine the effect of sialic acid binding for the infection of cultured cells under unfavorable conditions, and comparison of TGEV strains and mutants, as well as the avian coronavirus IBV concerning their dependence on the sialic acid binding activity.

**Methods:**

The infectivity of different viruses was analyzed by a plaque assay after adsorption times of 5, 20, and 60 min. Prior to infection, cultured cells were either treated with neuraminidase to deplete sialic acids from the cell surface, or mock-treated. In a second approach, pre-treatment of the virus with porcine intestinal mucin was performed, followed by the plaque assay after a 5 min adsorption time. A student's t-test was used to verify the significance of the results.

**Results:**

Desialylation of cells only had a minor effect on the infection by TGEV strain Purdue 46 when an adsorption period of 60 min was allowed for initiation of infection. However, when the adsorption time was reduced to 5 min the infectivity on desialylated cells decreased by more than 60%. A TGEV PUR46 mutant (HAD3) deficient in sialic acid binding showed a 77% lower titer than the parental virus after a 5 min adsorption time. After an adsorption time of 60 min the titer of HAD3 was 58% lower than that of TGEV PUR46. Another TGEV strain, TGEV Miller, and IBV Beaudette showed a reduction in infectivity after neuraminidase treatment of the cultured cells irrespective of the virion adsorption time.

**Conclusions:**

Our results suggest that the sialic acid binding activity facilitates the infection by TGEV under unfavorable environmental conditions. The dependence on the sialic acid binding activity for an efficient infection differs in the analyzed TGEV strains.

## Background

Enveloped viruses enter their target cells by a two step process [1]. The initial event is the attachment of the virion to the cell surface. Subsequently, the viral envelope fuses with the cellular membrane which enables the viral genome to get access to the cytoplasm. The fusion reaction may occur at the plasma membrane or, upon endocytotic uptake of the virion, at the endosomal membrane. The entry process requires the interaction of one or more viral surface proteins with cellular receptors. The binding to the cellular receptor mediates the attachment step and sets the stage for the subsequent fusion process. Several viruses have developed a strategy to recognize more than one surface structure of the target cell. The binding to some of these interaction partners may not be sufficient for the virus to proceed to the fusion step, but nevertheless it can support the entry process by making it more likely for the virus to find the actual cellular receptor.

TGEV is a porcine coronavirus that causes diarrhea in pigs of all ages [2]. Piglets even die from the infection unless they are protected by maternal antibodies. This enveloped virus with a positive-stranded RNA genome enters cells using the glycoprotein S for both attachment to the cell surface and for fusion of the viral membrane with the cellular membrane. The fusion activity of the S protein is induced only after interaction with a specific receptor on the surface of the target cell, porcine aminopeptidase N (pAPN) [3]. The S protein is not only able to bind to pAPN; it also has a sialic acid binding activity with a preference for N-glycolylneuraminic acid [4,5]. Interaction with sialylated macromolecules appears to be dispensable for infection of cultured cells but is believed to be important for the enteropathogenicity of the virus [6,7]. This is based on the finding that a single mutation in the S protein may result in the loss of both the sialic acid binding activity and the enteropathogenicity, whereas the mutant viruses can be propagated in cultured cells to the same titer as the parental virus [7,8]. This finding has been explained by environmental conditions in the intestine that make it more difficult for a microorganism to initiate an intestinal infection compared to an infection of cultured cells [9,10]. The intestinal epithelium is covered not only by a glycocalix layer but also by an even thicker layer of mucus [11]. As mucins are rich in sialic acids, they are interaction partners for TGEV and thus may help to penetrate the mucus layer and to get access to pAPN on the surface of the intestinal epithelial cells [9,10].

## Results and discussion

### Comparison of infectivities with and without neuraminidase treatment

**Figure 1:**
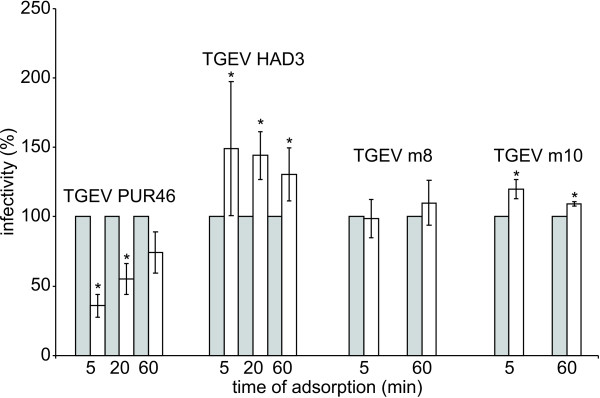
**Sialic acid dependent infection by TGEV PUR46**. The infectivity of parental virus (left columns) and the HAD3 mutant virus was determined for adsorption times of 5, 20 or 60 min, respectively. The m8 and m10 mutant viruses were analyzed for adsorption times of 5 and 60 minutes. Prior to infection, ST cells were incubated for 60 min with either PBS (grey columns) or PBS containing 50 mU of neuraminidase (white columns). All experiments were performed 4 times with standard deviations shown at the corresponding columns. Significant differences are marked with an asterisk (*, p-value < 0.05).

We tried to obtain experimental evidence for a role of the sialic acid binding activity of TGEV in the infection of cells under unfavorable conditions. For our analysis we used the Purdue strain of TGEV which was grown on swine testicular cells (ST) as described previously [5]. We analyzed the effect of desialylation of cells on infection by TGEV by a plaque assay [8]. In contrast to the regular plaque assay a neuraminidase treatment prior to infection was included to see a potential reduction in the number of plaques. To evaluate the optimal experimental setup, different neuraminidase concentrations were included in the first analysis (0, 50, 125, 250, 500, 1000, 1500 mU/ml; data not shown). A concentration of 250 mU/ml resulted in a significant inhibition of TGEV infection at an adsorption time of 5 min. As the cell culture appearance was not disturbed at this concentration, we decided to use this neuraminidase concentration of 250 mU/ml for subsequent experiments. As higher concentrations of the enzyme (up to 1.5 U/ml) gave no increase in virus inhibition, we concluded that sialic acids were removed to a satisfactory level. As shown in Figure 1 (columns designated TGEV PUR46) and table 1, pre-treatment of cells with neuraminidase from *Clostridium perfringens*, type V (250 mU/ml; Sigma) for 60 min reduced the infectivity of the parental virus by 26% when the adsorption time was 60 min. When the virus had only 5 min for adsorption, the infectivity on desialylated cells was reduced by 64%. Infection at 20 min adsorption time was in between these two values (reduction of 45%). This result is consistent with a previous work where binding of virions rather than infectivity was analyzed and where we have shown that desialylation of cultured cells reduces the binding of TGEV particles to these cells [5]. Our data indicate that the sialic acid binding activity increases the efficiency of infection at short adsorption times. This conclusion is supported by the finding that no reduction was observed when the hemagglutination deficient mutant HAD3 was subjected to such an analysis (Figure 1, table 1). This mutant has a point mutation in the S protein at position 209 (Leu-Pro) and was selected because of its deficiency in sialic acid binding [8]. Mutants m10 (deletion of 4 amino acids at position 146-149), and m8 (point mutation at amino acid 147) were analyzed in the same way for adsorption times of 5 and 60 min [6,7]. In fact, with these mutant viruses which are all deficient in sialic acid binding activity, pre-treatment of cells with neuraminidase even increased the infectivity. This increase was significant for mutants HAD3 and m10 with a p-value below 0.05 (table 1). In a previous publication we concluded that binding of the S protein to pAPN is even more efficient after neuraminidase treatment as sialic acid depletion on pAPN facilitates the protein binding [5]. It is possible that this effect makes it is easier for the mutants to get access to the cellular receptor pAPN and to bind to the specific binding site after neuraminidase treatment of the cell culture. In contrast, in the porcine intestine the binding of the mutants to pAPN could be less efficient because of the presence of sialic acids. For m8 and m10 it was reported that their enteropathogenicity is markedly reduced [6,7]. Therefore, a less efficient binding to the cellular receptor pAPN in vivo because of a deficiency in sialic acid binding could be one explanation for this reduced enteropathogenicity.

**Table 1:** Infectivities of virus strains and mutants after neuraminidase treatment of the cells

	Virus adsorption time		
	5 min	20 min	60 min
TGEV PUR46	35.8% (*, 4)	55.0% (*, 4)	74.4% (4)

TGEV HAD3	149.1% (*, 4)	144.1% (*, 4)	130.7% (*, 4)

TGEV m8	98.8% (4)	not determined	110.0% (4)

TGEV m10	119.9% (*, 4)	not determined	109.3% (*, 4)

TGEV Miller	67.7% (*, 3)	76.9% (*, 3)	74.0% (*, 3)

IBV Beaudette	66.6% (*, 3)	not determined	53.3% (*, 3)

The significance of the value is indicated by an asterisk, the number of independent experiments used for calculation is indicated in brackets.

**Figure 2:**
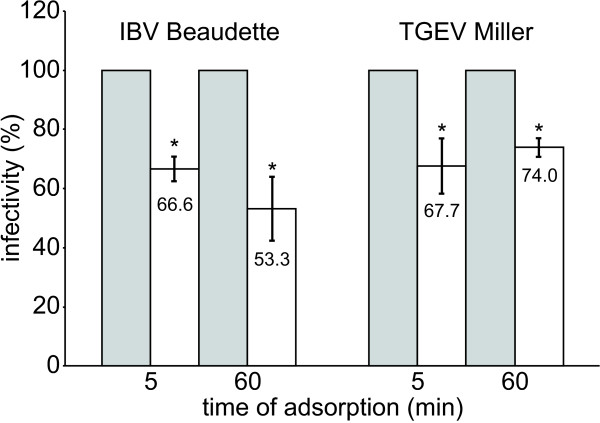
**Sialic acid dependent infection by IBV Beaudette and TGEV Miller at short and long adsorption times**. The infectivity of IBV Beaudette and TGEV Miller was determined at adsorption times of 5 or 60 min, respectively. Prior to infection, Vero cells (for IBV) and ST cells (for TGEV) were incubated for 60 min with either PBS (grey columns) or PBS containing 50 mU of neuraminidase (white columns). The experiments were performed 3 times. Standard deviations are indicated. Significant differences are marked with an asterisk (*, p-value < 0.05).

To include another TGEV strain in our study we analyzed the Miller strain [12]. This strain was mainly passaged in vivo, has an enteric and respiratory tropism like the Purdue strain and is virulent in swine [12,13]. As shown in Figure 2 and table 1, the Miller strain showed a reduction in infectivity after neuraminidase pre-treatment of the cells. This reduction is in the same range irrespective of the virion adsorption time (32% reduction for 5 min, 23% reduction for 20 min (table 1), 26% reduction for 60 min adsorption time).

Recently we have shown that infectious bronchitis virus (IBV), an avian coronavirus, uses sialic acid as a receptor determinant for infection of both cultured cells and tracheal organ cultures [14,15]. We were interested to know whether this virus also shows differences in the dependence on the sialic acid binding activity at short and long adsorption times. As shown in Figure 2 and table 1, when analyzed in the same way as TGEV, the Beaudette strain of IBV showed a reduction of the infectivity after pre-treatment of cells with neuraminidase. Similar to the result obtained with the Miller strain, the reduction was irrespective of a long or short adsorption time. After 60 min adsorption the infectivity of IBV was reduced by 47% and after 5 min adsorption time it was reduced by 33%. Taken together, our results indicate that the Miller strain of TGEV rather resembles IBV than the Purdue strain of TGEV as far as the sialic acid dependence of infection is concerned.

### Comparison of early and late infectivity

**Figure 3:**
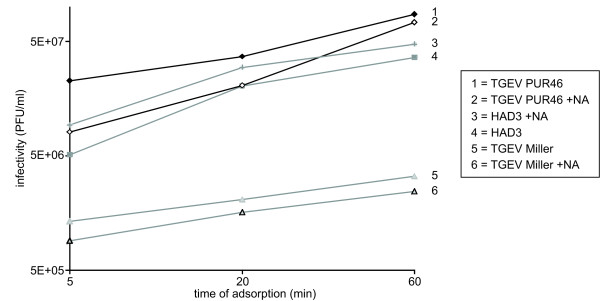
**Comparison of early and late infectivity between TGEV PUR46, HAD3 and TGEV Miller**. The infectious titers of TGEV PUR46, HAD3 and TGEV Miller were calculated at adsorption times of 5, 20 and 60 min, respectively. Infectivity was expressed in plaque forming units per ml (PFU/ml). Prior to infection, ST cells were incubated with either PBS (1, 4, 5) or PBS containing 50 mU of neuraminidase (2, 3, 6).

The infectious titer at three different time points (5, 20, 60 min) with and without neuraminidase treatment was calculated. Figure 3 shows a mean value out of 4 different experiments for TGEV PUR46, and HAD3, and out of 3 different experiments for TGEV Miller. Highest titers (2.27 × 10^7 ^PFU/ml at 5 min, 3.71 × 10^7 ^PFU/ml at 20 min, and 8.63 × 10^7 ^PFU/ml at 60 min) were obtained for TGEV PUR46 that increased over time. After neuraminidase treatment the titers decreased significantly for the first two time points (8.13 × 10^6 ^PFU/ml at 5 min, 2.06 × 10^7 ^PFU/ml at 20 min, and 7.33 × 10^7 ^PFU/ml at 60 min). Infectious titers for the hemagglutination deficient mutant HAD3 were 5.14 × 10^6 ^PFU/ml at 5 min adsorption time, 2.05 × 10^7 ^PFU/ml at 20 min, and 3.64 × 10^7 ^PFU/ml at 60 min. After neuraminidase treatment titers increased (9.30 × 10^6 ^PFU/ml at 5 min, 2.96 × 10^7 ^PFU/ml at 20 min, and 4.74 × 10^7 ^PFU/ml at 60 min). For the TGEV Miller strain infectious titers in cell culture were more than 10fold lower than for the TGEV Purdue strain (1.34 × 10^6 ^PFU/ml at 5 min, 2.09 × 10^6 ^PFU/ml at 20 min, 3.34 × 10^6 ^PFU/ml at 60 min). After neuraminidase treatment the titers of TGEV Miller decreased (9.08 × 10^5 ^PFU/ml at 5 min, 1.61 × 10^6 ^PFU/ml at 20 min, and 2.44 × 10^6 ^PFU/ml at 60 min). The differences in the titers between early (5 min) and late (60 min) infectivity of TGEV PUR46, HAD3, and TGEV Miller were statistically significant (with p < 0.05). The higher titers of TGEV PUR46 show that this virus strain is more cell culture adapted than the Miller strain. The early infectivity (5 min adsorption time) of HAD3 is about 22.6% of the value for TGEV PUR46 at this time point (with p = 0.014). However, the late infectivity (60 min) of HAD3 is about 42.2% of the value for TGEV PUR46 (with p = 0.021). This higher difference between TGEV PUR46 and HAD3 in early infectivity could be explained by the additional use of sialic acids for adsorption by TGEV PUR46. At short adsorption times, the importance of sialic acid binding for infectivity is more pronounced than at longer adsorption times when binding to the cellular receptor pAPN compensated this phenomenon.

### Reduction of TGEV infectivity by porcine intestinal mucins

**Figure 4:**
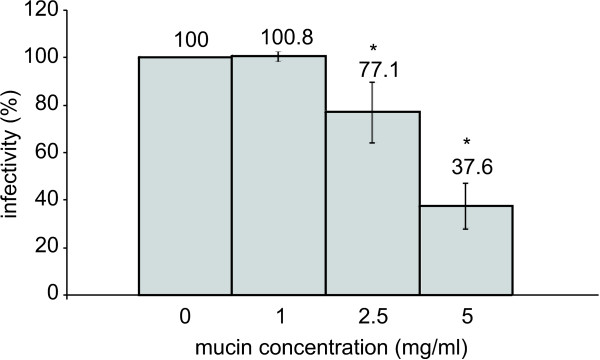
**Mucin-dependent infection of TGEV PUR46**. Prior to infection, TGEV PUR46 was treated with the indicated mucin concentrations for 30 min at room temperature. After a 5 min adsorption time of the virus-mucin-mixture a plaque assay was performed. Plaque reduction by mucin treatment was calculated out of 2 independent experiments with quadruplicates. Standard deviations are indicated. Significant differences are marked with an asterisk (*, p-value < 0.05).

To find out if the sialic acid binding activity of the TGEV S protein can be inhibited by porcine intestinal mucins which are rich in sialic acids we performed a plaque assay after incubation of TGEV PUR46 with different mucin concentrations as described in the material and methods section. Figure 4 shows that TGEV infectivity was inhibited by the mucin in a concentration dependent manner. Preincubation of the parental virus with 5 mg mucin/ml reduced its infectivity by 62% when the virus adsorption time was 5 min. Previous studies have shown that binding to these porcine intestinal mucins inhibited hemagglutination by TGEV [16]. Thus, sialic acid binding by the TGEV S protein appears to be inhibited by interaction with the tested mucin.

## Conclusions

From these results, the picture arises that coronaviruses have developed different strategies to make use of the sialic acid binding activity. These coronaviruses can be differentiated in three distinct groups. TGEV PUR46 as a representative of the first group recognizes pAPN so efficiently that the contribution of the sialic acid binding activity to the infection of cultured cells has remained unrecognized so far. Binding to pAPN is independent from sialic acids. It even appears to be more efficient when less sialic acid is present on pAPN itself [5]. Interaction with pAPN appears to be a slower process compared to sialic acid binding. Thus, effects produced by sialic acid binding can be recognized at short adsorption times when pAPN binding is still incomplete. Only by reducing the adsorption time we could show that the binding to sialoglycoconjugates on the cell surface increases the efficiency of TGEV PUR46 to infect cultured cells. For this strain the sialic acid binding activity may be required only to survive under unfavorable conditions as encountered in the intestinal tract. Interestingly, the Miller strain of TGEV did not show this significant difference in the infection efficiency between long and short adsorption times on neuraminidase-treated cells that was observed with the Purdue strain. There appear to be gradual differences in the importance of the sialic acid binding activity for different TGEV strains. The Miller strain of TGEV resembles IBV in its dependence on the sialic acid binding activity. These two viruses represent the second group of coronaviruses concerning their sialic acid binding activity. Pre-treatment of various cell types with neuraminidase has been shown to reduce the sensitivity to infection by different strains of IBV [14,15]. A protein receptor has so far not been identified for this avian coronavirus. The presence of such a receptor cannot be excluded. The third group of coronaviruses with sialic acid binding activity is represented by viruses like bovine coronavirus (BCoV) and human coronavirus OC43 (HCoV-OC43) [17,18]. These viruses resemble influenza C virus not only because of their preference for N-acetyl-9-O-acetylneuraminic acid but also because they contain an acetylesterase that functions as a receptor-destroying enzyme. The presence of this enzyme and the preference for a less common type of sialic acid are consistent with a higher affinity for the respective type of sialic acid. On the other hand, IBV which lacks a receptor-destroying enzyme requires a larger amount of sialic acid on the cell surfaces than do Sendai virus or influenza viruses and thus has a lower affinity for sialic acid than the latter viruses which contain a neuraminidase as a receptor-destroying enzyme [14]. These three groups of coronaviruses with sialic acid binding activity may represent different stages in an evolutionary process of using sialic acid for infection: i, acquisition of a sialic acid binding activity for increasing the efficiency of infection under unfavorable conditions (TGEV-Purdue); ii, modulation of the binding activity to exploit sialic acid as a general receptor determinant for attachment to target cells (TGEV-Miller, IBV); iii, further increase of the attachment efficiency by acquiring an enzyme that inactivates sialoglycoconjugates that prevent the spread of infection (BCoV, HCoV-OC43). Coronaviruses are characterized by one of the highest recombination frequencies among RNA viruses. In this way, they can acquire new properties by recombination events. An ancestral coronavirus might have had only a single receptor binding activity e.g. to a protein receptor and therefore only a single organ tropism e.g. the respiratory tract. By recombination events this virus could have acquired a sialic acid binding activity. Thus, such a virus should have a broadened tissue tropism e.g. additionally for the intestinal tract and increased chances to survive. As in the case for TGEV (with gradually differences), for such a virus the sialic acid binding activity may be an accessory function that renders the virus more efficient. Through further recombination events a receptor-destroying enzyme could have been acquired and a higher binding affinity to sialic acids could have been evolved. Such a virus, like BCoV, resembles influenza viruses. The gene for the receptor-destroying enzyme could have been incorporated in the coronavirus genome during coinfections of the same cell with coronaviruses and influenza viruses.

## Materials and methods

### Cell lines and virus strains

ST (swine testicular) and Vero cells were grown in Dulbecco's modified Eagle medium supplemented with fetal calf serum (10% for ST, 5% for Vero cells).

The Purdue strain of TGEV (PUR46-MAD) [19] was used throughout this study. Stock virus was propagated on ST cells. After incubation for 20 to 24 h at 37°C, the supernatant was harvested, clarified by centrifugation, and stored at -80°C after the addition of 1% fetal calf serum. The hemagglutination deficient mutant HAD3 (point mutation in the S protein at position 209 (Leu-Pro)) was selected for a deficiency in sialic acid binding [8]. Mutants m10 (deletion of 4 amino acids at position 146-149), and m8 (point mutation at amino acid 147 (Cys-Arg)) originally derived from the TGEV Purdue-115 strain [6,7]. The Miller strain of TGEV [12] was harvested after incubation for 48 h at 37°C on ST cells. Propagation of the Beaudette strain of IBV on Vero cells has been described recently [14].

### Neuraminidase treatment

Prior to infection, cells were either treated with 200 μl neuraminidase from *Clostridium perfringens*, type V (Sigma-Aldrich, St. Louis, Missouri, USA) diluted in PBS, or mock-treated, for 1 h at 37°C. For comparison between all tested viruses a concentration of 250 mU/ml was used. To compare the effect of different neuraminidase concentrations on the infection by TGEV PUR46, the neuraminidase was used in a concentration range of 50 mU/ml to 1.5 U/ml.

### Plaque reduction assay for TGEV and TGEV mutants

The effect of desialylated cells on TGEV infection was analyzed by a plaque assay [8]. ST cells were either treated with neuraminidase (see above), or mock-treated, for 1 h at 37°C. After washing, the cells were infected with TGEV PUR46, TGEV mutants HAD3, m10, and m8, or TGEV Miller. The reduction of plaques by neuraminidase treatment was calculated as a mean value out of 4 (3 for TGEV Miller) independent experiments.

### Plaque reduction assay for IBV Beaudette

The Plaque reduction assay for IBV Beaudette was performed on Vero cells as described previously [20]. The plaque reduction was calculated as a mean value out of 3 independent experiments.

### Mucin treatment

Virus was diluted in medium and incubated with different concentrations of porcine intestinal mucin (0, 1, 2.5, 5 mg/ml) for 30 min at room temperature [16]. ST cells were washed and incubated for 5 min at 37°C with these samples. Subsequently, a plaque assay was performed as described above. Plaque reduction by mucin treatment was calculated as a mean value out of 2 independent experiments with quadruplicates.

### Calculation methods

P-values were calculated in Microsoft Office Excel 2007 by using the student's t-test (single sided, type one). P < 0.05 was considered to indicate a statistically significant difference.

## Competing interests

The authors declare that they have no competing interests.

## Authors' contributions

CSW carried out the design of the study, analyzed the data, performed the statistical analysis and drafted the manuscript. SB participated in the analysis of the data and performed the plaque assays. CW participated in the IBV analysis. GH conceived of the study, participated in its design and coordination and helped to draft the manuscript. LE and HL participated in the design of the study. All authors read and approved the final manuscript.
